# Periodontal ligament stem cell tissue engineering scaffolds can guide and promote canine periodontal tissue regeneration

**DOI:** 10.3389/fvets.2024.1465879

**Published:** 2024-10-09

**Authors:** Pengxiu Dai, Guixiang Qi, Mingde Zhu, Qingjie Du, Keyi Wang, Yaxin Gao, Mengnan Li, Xiancheng Feng, Xinke Zhang

**Affiliations:** The College of Veterinary Medicine, Northwest Agriculture and Forestry University, Yangling, China

**Keywords:** periodontal disease, periodontal ligament stem cells, canine PDLSC tissue engineering scaffold, guide periodontal regeneration, periodontal repair

## Abstract

**Introduction:**

Periodontal disease, including gingivitis and periodontitis, is caused by dental plaque invading the periodontal tissues and is the most common oral disease. The basic treatment methods applied in the clinic can destroy dental plaque, smooth the root surface, and reduce local inflammation, but it is difficult to completely regenerate and rebuild the complex three-dimensional periodontal tissues. The rapid development of periodontal tissue engineering has led to the development of new methods for the treatment of periodontal disease. Periodontal ligament stem cells (PDLSCs) are key seed cells in periodontal tissue engineering, which can provide strong support for tissue regeneration. Meanwhile, an engineering scaffold constructed from biomaterials provides a three-dimensional space for the growth and function of seed cells and can form a tissue engineering complex with the seed cells to repair periodontal tissue, which can guide consequently enable true three-dimensional periodontal structure regeneration and functional restoration.

**Methods:**

This study established an effective way to isolate, culture, and identify canine PDLSCs. Using chitosan, β-glycerol phosphate, and biphasic calcium phosphate bone substitute as raw materials, a tissue engineering scaffold with good physical properties was prepared by freeze-drying method. Canine PDLSCs were co-cultured with the scaffolds to prepare canine PDLSC tissue engineering scaffolds with good biocompatibility *in vivo* and *in vitro*.

**Results and discussion:**

The canine PDLSC tissue engineering scaffold was transplanted into the single wall bone defect of the first mandibular molar tooth of the dog without causing inflammatory reactions, and the tissue compatibility was satisfactory. The cell-scaffold complex can increase the content of related growth factors and immunomodulatory factors in the tissues, reduce the content of proinflammatory factors, and prevent the growth of binding epithelium in the defect area, thus forming new bone and new periodontal ligaments in the defect area, promoting the repair of periodontal defects, and improving the therapeutic effect of guided regeneration.

## Graphical abstract

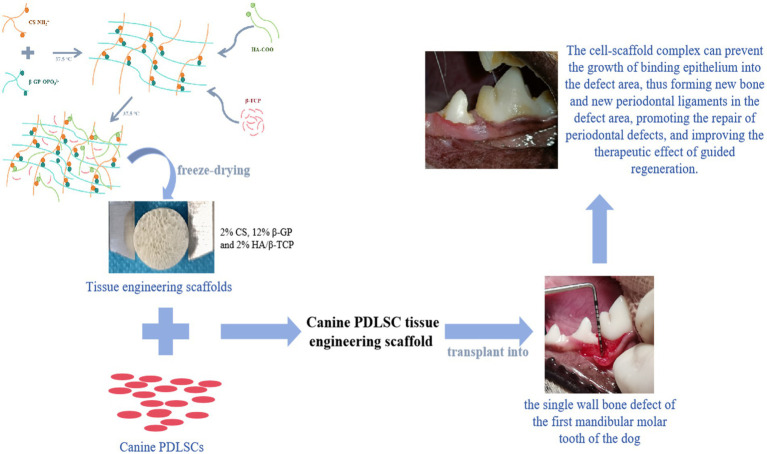

## Introduction

Periodontal disease is the most common oral disease in dogs. Adult dogs of all ages can suffer from periodontal disease to varying degrees. According to statistics, 80% of dogs over 2 years old suffer from periodontal disease (1, 2). As the disease progresses, periodontal disease can cause symptoms such as pain, difficulty eating, bad breath and salivation, and in severe cases, other systemic diseases such as gastrointestinal tract, cardiovascular and kidney can be secondary, increasing the overall inflammatory burden of the body (3). At the same time, periodontal ligament, alveolar bone and other supporting tissues will be damaged by periodontal disease and gradually lost.

At present, the basic treatment methods applied in the clinic can destroy dental plaque, smooth the root surface, and reduce local inflammation, but it is difficult to completely regenerate and rebuild the complex three-dimensional periodontal tissues (4, 5). During the healing process after basic treatment, the conjunctive epithelium attaches to the root surface and migrates along the root to the apex. The resulting long conjunctive epithelium blocks contact between the periodontal membrane fibers and the root surface. At this time, the gingival groove deepens, pathogenic microorganisms reattach and accumulate, and the disease easily recurs (5, 6). The ideal periodontal repair method should restore damaged periodontal structure and function; rebuild the three-dimensional structure of the alveolar bone, cementum and periodontal membrane; maintain the spatial position of periodontal tissue during repair and regeneration; promote new periodontal attachments; and ultimately restore the structure and function of normal periodontal tissue (7). Guided tissue regeneration (GTR) was proposed based on the theory of periodontal regeneration to help complete the regeneration and functional reconstruction periodontal tissue and to prevent the attachment of the neonatal junctional epithelium to the tooth root surface. GTR repairs and regenerates periodontal tissue structure by guiding specific cells to the right place. In this approach, a physical barrier membrane is placed over the periodontal defect between the soft tissue flap and the root surface, keeping the gingival epithelium away from the root surface and creating a protected space above the defect that allows periodontal membrane cells to refill the wound (8).

In recent years, the rapid development of periodontal tissue engineering has led to the development of new methods for the treatment of periodontal diseases. Periodontal ligament stem cells (PDLSCs) are key seed cells in periodontal tissue engineering. PDLSCs can proliferate, differentiate, and secrete a variety of cytokines during periodontal tissue repair, providing strong support for tissue regeneration (9). An engineering scaffold constructed from biomaterials provides a three-dimensional space for the growth and function of seed cells and can form a tissue engineering complex with the seed cells to repair periodontal tissue. Moreover, guided periodontal regeneration can prevent the conjunctive epithelium from attaching to the root surface, maintain the spatial position of periodontal tissue during regeneration, and consequently enable true three-dimensional periodontal structure regeneration and functional restoration (10).

In this study, canine PDLSCs were isolated and cultured *in vitro*, and their differentiation, proliferation ability and biological characteristics were assessed to establish an effective way to isolate and culture canine PDLSCs to obtain sufficient seed cells for periodontal tissue engineering. Moreover, chitosan (CS), *β*-glycerol phosphate (β-GP), and biphase calcium phosphate bone replacement material (HA/β-TCP) were used as raw materials to prepare scaffolds with different proportions via the freeze–drying method. The physical properties of the different scaffolds, such as morphology, pore size, porosity, compressive strength, swelling rate and degradation rate, were compared. Canine PDLSCs were cocultured with scaffolds to explore their biocompatibility *in vivo* and *in vitro*. On this basis, a single-wall bone defect model at the first mandibular molar of a dog was prepared, and a canine PDLSC tissue engineering scaffold was implanted into the periodontal defect to evaluate its effect in guiding periodontal regeneration, providing a reference and basis for the repair and regeneration of periodontal tissue in periodontal disease.

## Method

### Cell segregation

Canine retained deciduous teeth were collected, and periodontal tissue was isolated by surgical methods. The whole operation was performed with strictly adherence to aseptic principles and procedures for tooth extraction. The collected periodontal membrane tissues were cut and placed in a centrifuge tube on a superclean workbench, and type I collagenase (3 mg/mL) (Merck KGaA, SCR103, Darmstadt, Germany) solution and neutral protease (4 mg/mL) (Merck KGaA, COLLDISP-RO, Darmstadt, Germany) solution were added at a ratio of 1:1 (volume ratio). The sample was digested at 180 rpm at 37°C for 1.5 h in a shaker. Then, complete cell culture solution (*α*-MEM culture medium (Thermo Fisher Scientific, 12,571,063, Massachusetts, United States) containing 10% fetal bovine serum (Thermo Fisher Scientific, A5256701, Massachusetts, United States), 1% 100× penicillin–streptomycin solution and 0.25% anti-Myc mycoplasma scavenging reagent) was added to the centrifuge tube to terminate digestion, and the mixture was centrifuged at 1000 rpm for 5 min. The supernatant was discarded, fresh complete cell culture medium was added to resuspend the cell pellet, and the suspension was mixed by aspiration several times and inoculated into the culture dish. The cell density and state were observed under a microscope, and the cells were cultured in an incubator at 37°C and 5% CO_2_ and observed every other day. When 90% confluence was reached, the cells were passaged at a 1:3 ratio by trypsin digestion.

### Cell identification

Cells from the third passage were inoculated into 24-well plates. The medium was changed every other day, and cell counts were performed every 24 h. Cells from 3 wells were removed at a time, and the cells in each well were counted 6 times. According to the counting results, a cell growth curve was drawn with the average cell number as the ordinate and the culture time as the abscissa.

Cells from the third passage were cultured under standard conditions. When 90% confluence was observed under the microscope, the cells were stained with hematoxylin–eosin, Giemsa, and alkaline phosphatase. The cells were stained by immunofluorescence with vimentin, keratin primary antibody (Abcam, ab20346, ab8068, Cambridge, United Kingdom) and corresponding secondary antibody (Abcam, England).

Using CD31, CD44, CD45, CD73, CD90, CD105, CD166, CD11a, and STRO-1 antibodies (Abcam, ab28364, ab189524, ab40763, ab317462, ab92574, ab206419, ab235957, ab25383 and ab214086, Cambridge, United Kingdom) and control antibodies with FITC and PE fluorescent labels, the expression of molecular markers on the cell surface was measured via flow cytometry (Agilent, NovoCyte Penteon, California, United States).

Three-line induction differentiation kit (OriCell® Mesenchymal Stem Cell Osteogenic Induction Differentiation Kit, HUXMX-90021, OriCell® Mesenchymal Stem Cell Chondrogenic Induction Differentiation Kit, HUXMD-90041, OriCell® Mesenchymal Stem Cell Adipogenic Induction Differentiation Kit, HUXMX-90031, Cyagen Biosciences (Guangzhou) Co., Ltd., China) were used according to the manufacturer’s instructions to perform three-line induction differentiation of cells.

### Preparation of scaffolds

Chitosan (CS) (Merck KGaA, 419,419, Darmstadt, Germany) was dissolved in 0.1 M acetic acid to prepare CS solution. Sodium *β*-glycerol phosphate (*β*-GP) (Merck KGaA, 50,020, Darmstadt, Germany) was dissolved in ultrapure water to prepare *β*-GP solution.

The β-GP solution was added dropwise to the CS solution to prepare solutions with CS: *β*-GP mass ratios of 1:2, 1:3, 1:4, 1:5, 1:6, and 1:7. The w/v of chitosan (CS) was 2%, and the w/v of β-GP was 4, 6, 8, 10, 12, and 14%, respectively. After stirring well at room temperature for 15 min, 2 mL of each sample was taken and placed in a centrifuge tube and incubated in a 37.5°C water bath. The sample was removed every 1 min and inverted to observe the gel fluidity. The inverted 30 s nonflowing time was recorded as the gel formation time.

After the CS/*β*-GP gel was prepared, different amounts of HA/β-TCP (0, 2, 4%) (Merck KGaA, 677,418, 693,898, Darmstadt, Germany) were dispersed into the gel system. After constant-temperature magnetic stirring for 2 h, 1.5 mL of each sample was removed and placed in a water bath at 37.5°C to form a gel. The gel formation time was measured by the bottle rotation method. Samples in a stable gel state were prefrozen at −80°C for 24 h and vacuum freeze-dried for 12 h.

### Physical detection of the scaffolds

The prepared support was cut into cylinders of equal size (diameter of 7 mm and height of 15 mm) with flat upper and lower surfaces. The compressive strength of the support was tested by means of a Zwick 250 electronic universal testing machine (ZwickRoell, Universal Materials Testing Machine, ZWICK Z250, Germany). The calculation formula is $\sigma =\frac{4F}{\pi d^{2}}$ , where *σ* is the compressive strength (MPa) of the test support, F is the maximum compressive force (N) borne by the test support, and d is the diameter of the test support (mm). Four scaffold samples from each group were tested in parallel.

The prepared scaffolds were cut into round sheets (7 mm in diameter). Scanning electron microscopy (Carl Zeiss AG, Sigma300, Oberkochen, Germany) was used to observe the surface morphology and internal structure of the scaffold material, and the aperture size and porosity were calculated with ImageJ. The initial weight of the support was accurately determined and recorded as W_0_. The tissue engineering scaffold was subsequently immersed in a test tube filled with normal saline, which was sealed and placed in a 37.5°C water bath. The scaffold was removed at 24 h, the weight was recorded as W_S_. Four samples from each group were tested in parallel, and the swelling rate of the tissue engineering scaffold was calculated. The formula is $\mathrm{swelling ratio}=\frac{W_{S}-W_{0}}{W_{0}}$ . The initial weight of the support was accurately determined and recorded as W_1_. The tissue engineering scaffold was subsequently immersed in a test tube containing normal saline, which was sealed and placed in a 37.5°C water bath. Samples were removed at 4, 7, 14 and 21 days and dried for 10 h, and the weight was recorded as W_2_. Four parallel samples were tested for each group, after which the degradation rate of the scaffolds was calculated at different time points. The calculation formula is


$$\mathrm{degradation}\;\mathrm{rate}=\frac{W_{1}-W_{2}}{W_{1}}\times 100\%$$

### *In vitro* biocompatibility of the scaffolds

The prepared scaffolds were cut into cuboids (4 × 4 × 2 mm), placed in a closed space, and irradiated by a high-ozone ultraviolet disinfection lamp (185 nm/UVD) for disinfection and sterilization for 24 h. After soaking in high-resistance PBS for 3 h, the scaffolds were placed in cell culture medium for 24 h.

A suspension of 1.25 × 10^6^ cells/mL was inoculated onto a scaffold in a 96-well plate. The volume in each well was 100 μL, the cells were cultured in a cell incubator, and the medium was changed every other day. The scaffolds were removed on Days 1, 3, and 5 and transferred to a new 96-well plate. The number of cells attached to the scaffold and the number of cells attached to the bottom of the dish were determined by a CCK-8 kit (MedChemExpress, HY-K0301, New Jersey, United States), and the adhesion and proliferation of the cells on the scaffold were evaluated.

The cell membrane fluorescent dye CM-Dil (MedChemExpress, HY-D1028, New Jersey, United States) was used to label canine PDLSCs, and a CM-Dil-labeled canine PDLSC suspension was prepared. A 200 μL sample of cell suspension was taken up by a microsyringe and injected into the scaffold. Twelve hours later, 800 μL of cell medium was added to observe the adhesion and growth of the labeled cells on the scaffold on the 1st, 3rd and 5th days.

Fresh cell culture medium was added to the scaffold at 0.05 g/10 mL, and the mixture was incubated in the cell incubator for 24 h. Then, the extract was collected and stored for later use. A total of 1.5 × 10^5^ cells/mL were inoculated into a 6-well plate. After cell growth to 100% confluence, a parallel line was drawn in the Petri dish using a 10 μL pipette, the cell debris was gently rinsed with PBS, and scaffold extract was added. Cell migration was observed at 24 h and 48 h, and the migration area was measured and analyzed by ImageJ.

### Evaluation of scaffold biocompatibility *in vivo*

All of the dogs (Beagle, 2–3 years old, 8–12 kg, male or female) were reared, obtained, and housed in accordance with our institute’s laboratory animal requirements, the dogs were kept in cages in a feeding room without purification equipment at a temperature of 18–25°C, humidity of 40–60%, airflow value of 0.13–0.18 m/s, ventilation rate of 10–20 times per hour, light normal, noise below 60 dB.

The experimental dogs were injected intravenously with propofol (3 ~ 6 mg/kg) (Xi ‘an Libon Pharmaceutical Co., Ltd., China) to induce anesthesia before surgery, endotracheal intubation was performed to protect the airway, and a respiratory anesthesia machine was connected. The isoflurane (Shenzhen Reward Life Technology Co., Ltd., China) and oxygen flows were adjusted, and when anesthesia was stably maintained, the body fluids were supplemented by intravenous infusion of 5% glucose. The dog was prone, from the shoulder blades to the final ribs, within 8 cm on both sides of the spine, for routine skin preparation. Four centimeters from the spine, a 2 cm long skin incision was made parallel to the spine to bluntly separate the subcutaneous tissue. Canine PDLSC tissue engineering scaffolds (experimental group, *n* = 5) and cell-free scaffolds (control group, *n* = 5) were implanted into the incisions on both sides of the spine, with the side chosen at random. The skin was sutured, and the incision was covered with sterile gauze. Amoxicillin and clavulanate potassium were injected daily for 3 consecutive days to prevent postoperative infection, and the stitches were removed on the 7th day after surgery.

Blood was collected from the test dogs 1 day before subcutaneous implantation and 21 days after implantation for biochemical detection. Complications and adverse events during postoperative healing in all dogs were recorded. Wound healing was evaluated on Days 7, 14 and 21 after subcutaneous implantation according to the Vancouver Scar Scale (11). The subcutaneous tissue containing the complex (sample at least 0.5 cm larger than the scaffold boundary) was removed on the 21st day after surgery, and paraffin sections were prepared and stained with hematoxylin–eosin (HE). The biocompatibility of the canine PDLSC tissue engineered scaffold was evaluated.

### Evaluation of the effectiveness of periodontal defect repair and regeneration

All of the dogs (Beagle, 2–3 years old, 8–12 kg, male or female) were reared, obtained, and housed in accordance with our institute’s laboratory animal requirements, the dogs were kept in cages in a feeding room without purification equipment at a temperature of 18–25°C, humidity of 40–60%, airflow value of 0.13–0.18 m/s, ventilation rate of 10–20 times per hour, light normal, noise below 60 dB.

The experimental dogs were injected intravenously with propofol (3 ~ 6 mg/kg) (Xi ‘an Libon Pharmaceutical Co., Ltd., China) to induce anesthesia before surgery, endotracheal intubation was performed to protect the airway, and a respiratory anesthesia machine was connected. The isoflurane (Shenzhen Reward Life Technology Co., Ltd., China) and oxygen flows were adjusted, and when anesthesia was stably maintained, the body fluids were supplemented by intravenous infusion of 5% glucose. Inferior alveolar nerve conduction anesthesia was induced by an injection of lidocaine (4 mg/kg). The operation was subsequently carried out in strict accordance with the established surgical protocol and aseptic rules. During the operation, the central nervous system, cardiovascular system and respiratory system were monitored, and temperature changes were recorded to monitor the animal’s status. Before the start of treatment, the oral cavity was rinsed with 0.12% chlorhexidine gluconate solution, and oral radiographs were taken to record the tooth status.

A horizontal incision parallel to the gingival margin was made along the lingual side of the first molar and the buccal gingival margin, and a vertical incision was made along the fourth premolar to cut the gingiva, lift the periodontal tissue flap, and fully expose the alveolar bone and periodontal space. A high-speed drill was used to remove the alveolar bone between the fourth premolar and the first molar. The periodontal ligament, alveolar bone and cementum were completely destroyed to form a single-wall bone pocket (6 mm × 2 mm, depth × proximal and distal width) to establish a single-wall bone defect model at the mandibular first tooth. In accordance with the protocol for the random group, the materials for each group [canine PDLSC tissue engineering scaffold group (*n* = 8) and scaffold group (*n* = 8)] were transplanted into the periodontal defect site close to the surrounding tissues for guided regeneration. In the control group (*n* = 8), the gingival tissue flap was sutured without any transplantation treatment. For 3 consecutive days after the operation, amoxicillin and clavulanate potassium were subcutaneously injected, and 0.12% chlorhexidine gluconate solution was used to flush the mouth to prevent intraoperative infection. Oral procedures were limited to avoid exerting pressure or tension on the sutures. Wound healing was observed at 2, 4 and 8 weeks after the operation. At the 4th week after surgery, the grafted tissue was collected, extraction buffer precooled at 4°C was added, and the tissue sample was homogenized, subjected to one freeze–thaw cycle, ultrasonicated for 10 min, incubated at 4°C for 1 h, and centrifuged at 120,000 × g for 10 min. Relevant factors in the supernatant were detected via an ELISA kit. At the 8th week after the operation, the tissue from the dog transplantation site was collected, fresh non-decalcified tissue specimens were fixed and dehydrated, impregnated with light curing resin, and then sawed, and hard tissue sections 8 ~ 10 μm in thickness were obtained by grinding the slices (EXAKT hard tissue cutting and grinding system). Hematoxylin and eosin (HE) staining was used to observe periodontal tissue repair and regeneration, Masson staining was used to observe periodontal membrane fiber attachment, and toluidine blue staining was used to observe new bone formation. The JE (junctional epithelium), NBH (new bone height), NBA (new bone area) and PDL (periodontal ligament regeneration) were evaluated.

### Statistical analysis

One-way analysis of variance (ANOVA) and Two-factor Analysis of Variance (Two-way ANOVA) were used for the statistical comparisons among groups. The tests were performed using IBM SPSS Statistics 25 software (SPSS Inc., Chicago, IL, United States).

## Results

### Results of cell isolation and culture

**Figure 1:**
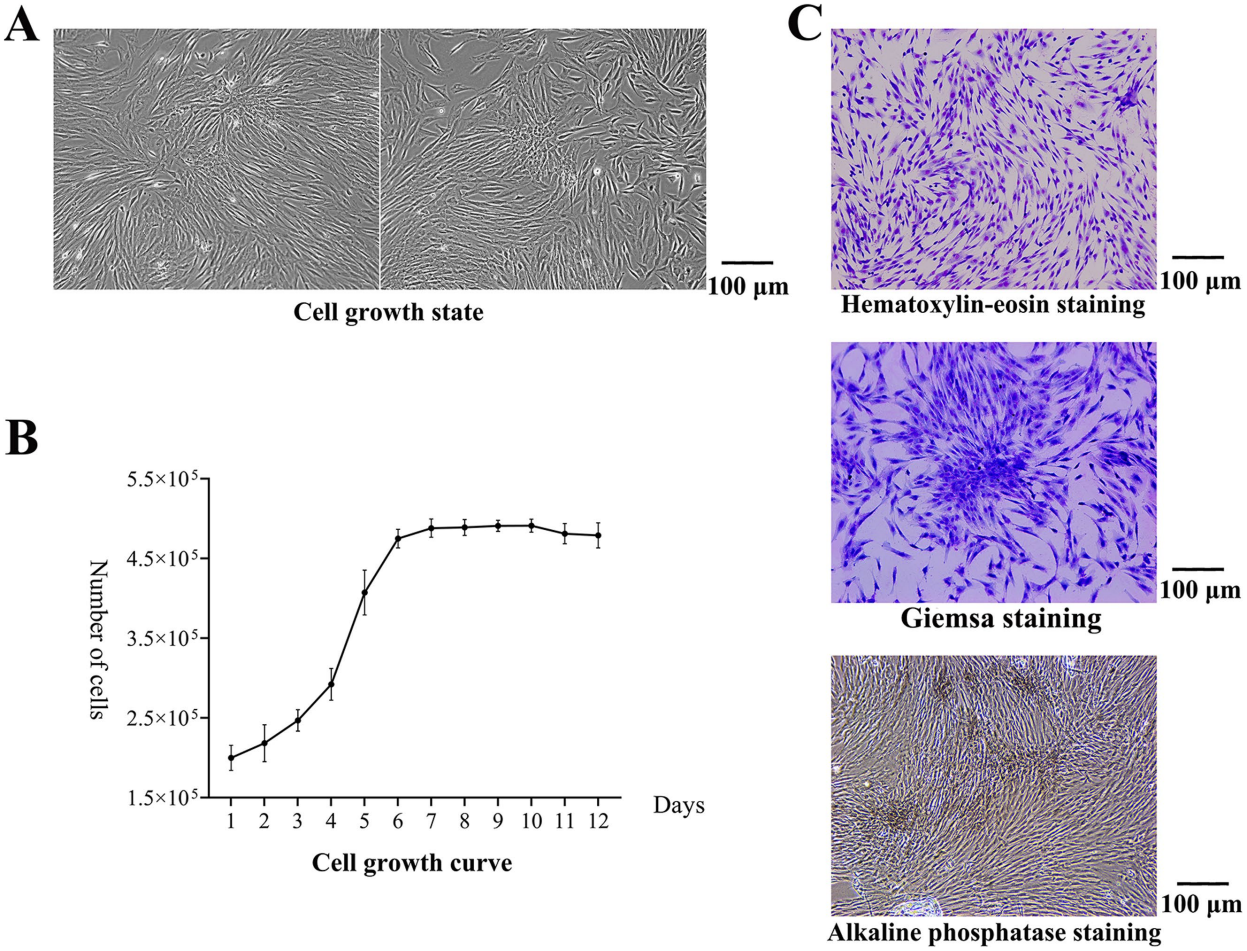
Results of cell isolation and culture. **(A)** Cell growth state. The adherent cells covered the entire bottom of the dish. **(B)** The cell growth curve. The cells entered the exponential growth phase on the 3rd day. **(C)** After hematoxylin–eosin staining, the cells were spindle-shaped and adhered to the wall. After Giemsa staining, the colonies were observed. The boundaries between the central cells of the colonies were not clear, and the surrounding cells were tightly arranged radially. After alkaline phosphatase staining, brown and gray particles were observed among the closely packed cells, and more particles were found in the clonal colonies.

After 3–5 days of culture, the cells began to adhere to the wall, and there was outward migration and growth around the tissue blocks. With increasing culture time, the number of adherent cells increased continuously, and the adherent cells covered the entire bottom of the dish (Figure 1A). The primary cultured cells were passaged twice, the cells in the P3 generation were selected to construct the cell growth curve, and the cells entered the exponential growth phase on the 3rd day (Figure 1B). After hematoxylin–eosin staining, the cells were spindle-shaped and adhered to the wall. After Giemsa staining, the colonies were observed. The boundaries between the central cells of the colonies were not clear, and the surrounding cells were tightly arranged radially. After alkaline phosphatase staining, brown and gray particles were observed among the closely packed cells, and more particles were found in the clonal colonies, indicating positive expression of alkaline phosphatase (Figure 1C).

### Cell identification

**Figure 2:**
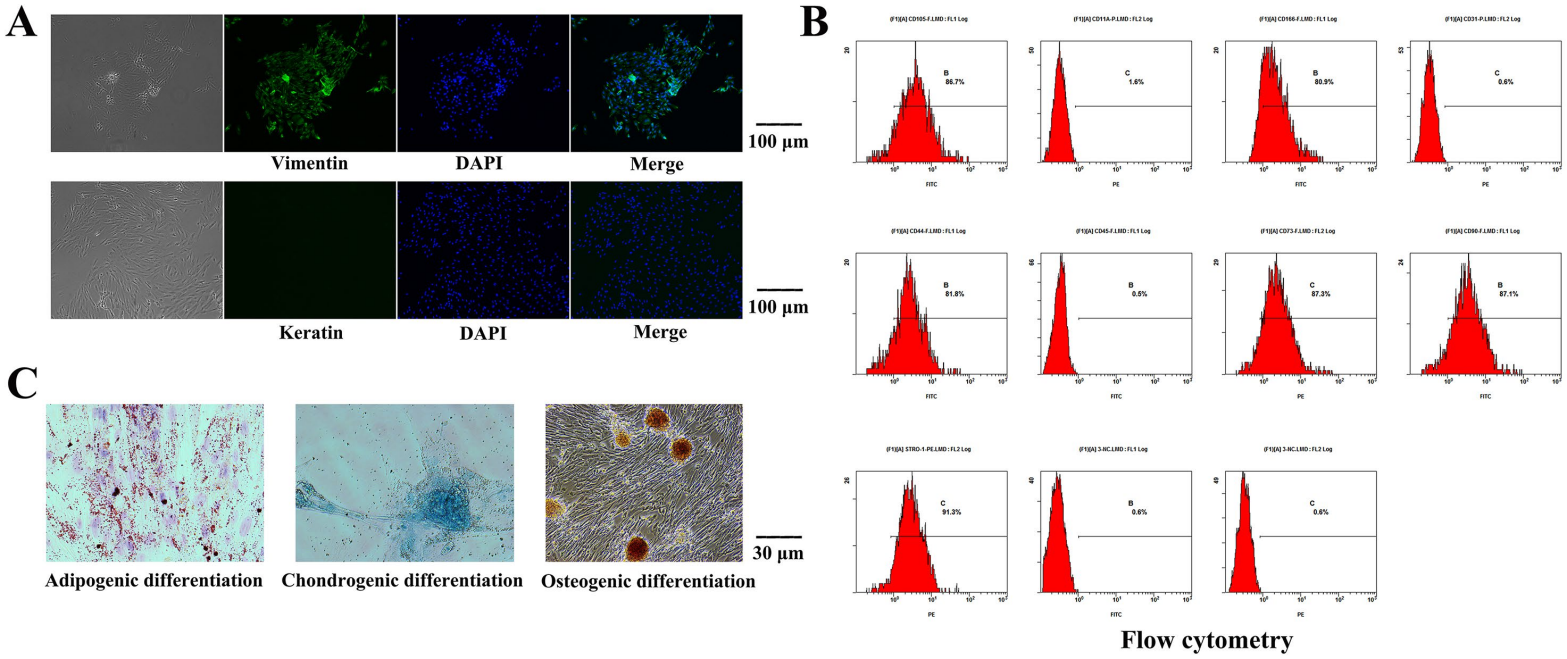
Results of Cell identification. **(A)** The isolated cells were subjected to immunofluorescence staining. The nucleus was blue, while no red fluorescence was observed to indicate keratin. The isolated cells were positive for vimentin expression. **(B)** The expression levels of STRO-1, CD73, CD90, CD105, CD44 and CD166 were 91.3, 87.3, 87.1, 86.7, 81.8 and 80.9%, respectively, according to flow cytometry. CD11a, CD31 and CD45 were not expressed. **(C)** Lipid-induced differentiation was performed on the isolated cells, lipid droplet particles were observed upon staining with oil red O. The isolated cells were differentiated by osteogenic induction, and alizarin red staining revealed a large area of tangerine-mineralized nodules. The isolated cells were subjected to chondroblast differentiation induction, and the cells were staining positive for Alcian blue.

The isolated cells were subjected to vimentin immunofluorescence staining. Under an inverted microscope, vimentin was labeled with green fluorescence, and the nucleus was blue, while no red fluorescence was observed to indicate keratin (Figure 2A). The isolated cells were positive for vimentin expression and negative for keratin expression, which proved that the cells were of mesodermal origin without epithelial cell contamination.

The expression levels of STRO-1, CD73, CD90, CD105, CD44 and CD166 were 91.3, 87.3, 87.1, 86.7, 81.8 and 80.9%, respectively, according to flow cytometry. CD11a, CD31 and CD45 were not expressed (Figure 2B). Lipid-induced differentiation was performed on the isolated cells. Bright red grape beaded or paver-like lipid droplet particles were observed upon staining with oil red O, and some of the lipid droplet particles were fused with each other. The isolated cells were differentiated by osteogenic induction, and alizarin red staining revealed a large area of tangerine-mineralized nodules. The isolated cells were subjected to chondroblast differentiation induction, and the cells were stained by Alcian blue (Figure 2C).

### Morphological characteristics of the scaffolds

**Figure 3:**
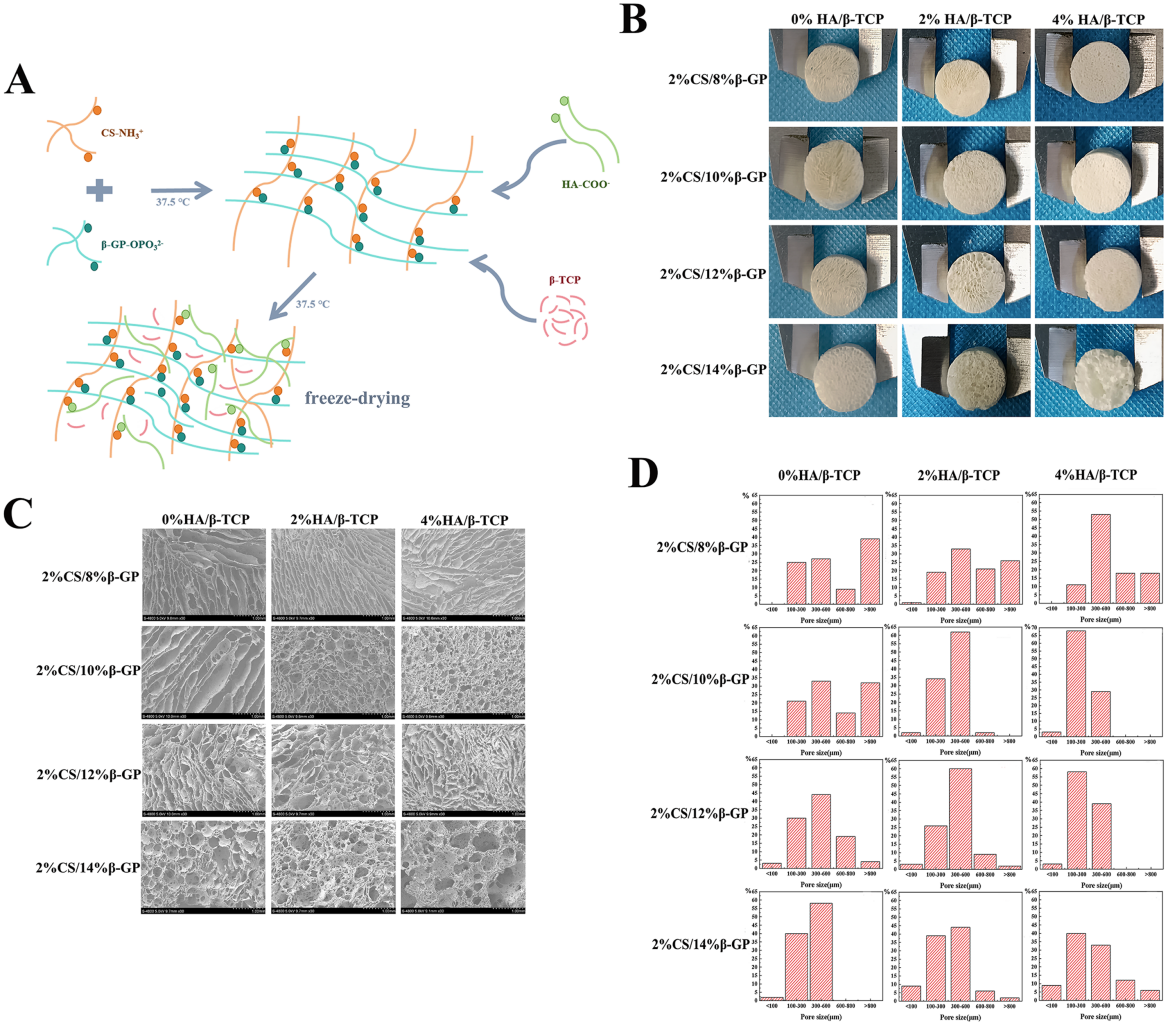
Morphological characteristics of the scaffolds. **(A)** Using chitosan (CS), *β*-glycerol phosphate (β-GP) and biphase calcium phosphate bone substitute (HA/β-TCP) as raw materials, a tissue engineering scaffold was prepared by freeze-drying method. **(B)** Twelve groups of scaffolds were prepared after vacuum freeze-drying, all of which were cylindrical with regular external micromorphology. **(C)** The 12 groups of scaffolds had a three-dimensional spatial structure with interconnecting pores. With increasing β-GP content, the pores changed from a loose lamellar structure to a stable honeycomb structure, and the surface roughness increased after the addition of HA/β-TCP. **(D)** The porosity of the scaffolds was calculated using ImageJ, and a pore distribution map was constructed.

During testing of the CS gel matrix, the mixture of 2% CS/4% *β*-GP did not form a gel matrix at 37.5°C, and 2% CS/6% *β*-GP formed a gel matrix at approximately 50 min; however, the gel state was unstable, and flow occurred after inversion for 1 min. The solutions containing 2% CS/8, 10, 12, and 14% *β*-GP formed a gel matrix within 3 to 10 min at 37.5°C, and the gel state was stable without fluidity after inversion. HA/β-TCP particles were uniformly dispersed into the CS gel matrix, and different amounts of HA/*β*-TCP were added to 2% CS/8, 10, 12, and 14% β-GP. Twelve groups of scaffolds were prepared after vacuum freeze-drying, all of which were cylindrical with regular external micromorphology (Figures 3A,B). The cross-sectional structure of the scaffolds was subsequently observed via field emission scanning electron microscopy. The 12 groups of scaffolds had a three-dimensional spatial structure with interconnecting pores. With increasing *β*-GP content, the pores changed from a loose lamellar structure to a stable honeycomb structure, and the surface roughness increased after the addition of HA/*β*-TCP (Figure 3C).

The porosity of the scaffolds was calculated using ImageJ, and a pore distribution map was constructed (Figure 3D). When HA/*β*-TCP was not added, the number of pores larger than 800 μm decreased with increasing *β*-GP content. With the scaffold with 14% *β*-GP had the highest proportion of 300 to 600 μm pores, followed by that of the scaffold with 12% *β*-GP, and there were pores smaller than 100 μm in these two groups; moreover, the appearance of these small pores increased the internal connectivity within the scaffold. When 2% HA/β-TCP was added to the 12% β-GP scaffold, 60% of the scaffold pores were 300–600 μm in diameter pores, and 2% were less than 100 μm in diameter. When 4% HA/β-TCP was added to the 8% β-GP scaffold, pore sizes of 300 ~ 600 μm were the most common. When 4% HA/*β*-TCP was added to the 10% β-GP, 12% β-GP and 14% β-GP scaffolds, 68, 58 and 33% of the pores were 100–300 μm in diameter, respectively.

### Compressive properties and porosity of the scaffolds

**Figure 4:**
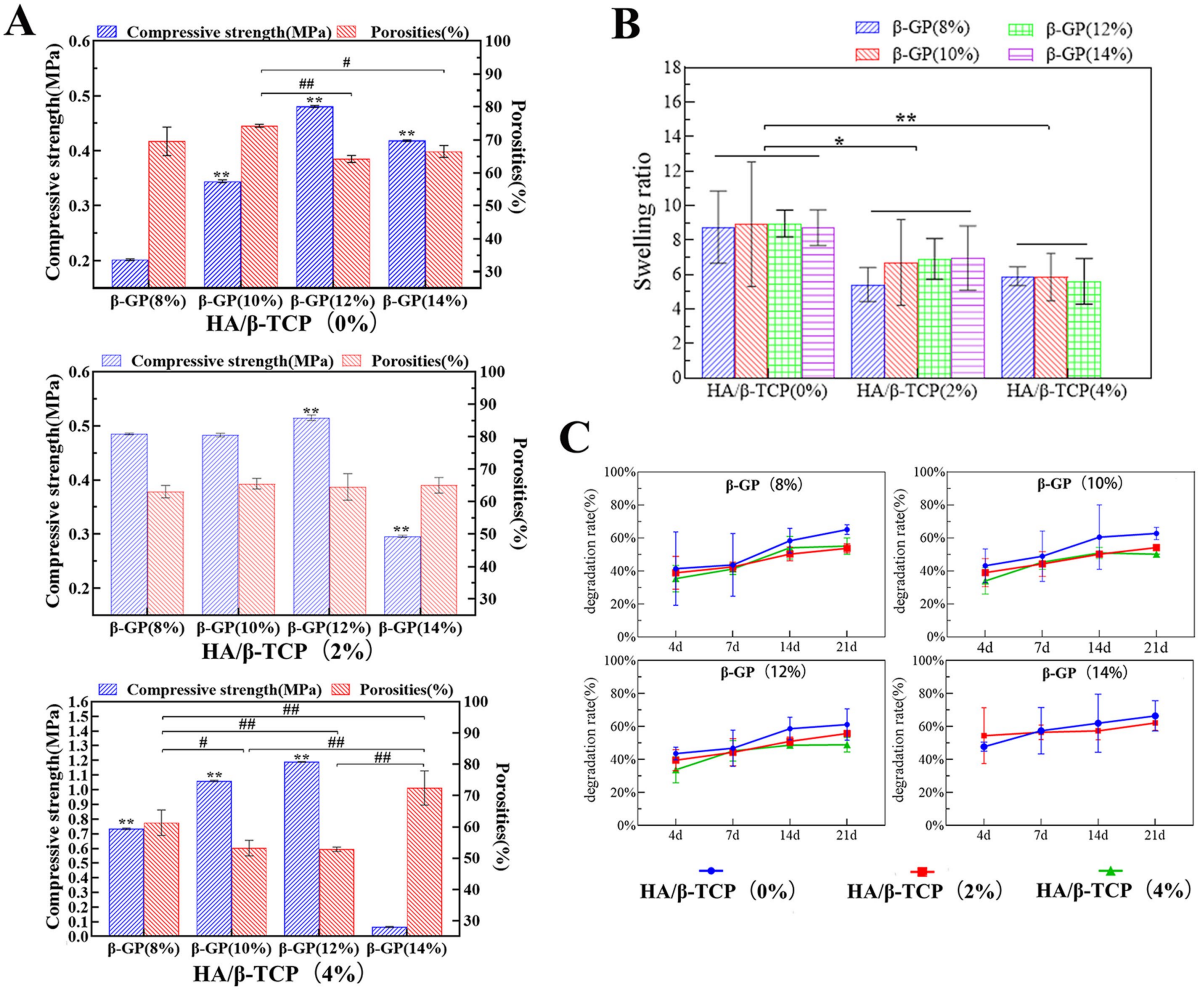
Physical performance test results of the scaffolds. **(A)** Compressive properties and porosity of the scaffolds. The scaffold prepared with 12% β-GP and 2% HA/β-TCP was most in line with practical needs. **(B)** Results of the swelling performance test. When the HA/β-TCP content was constant, the β-GP content had no significant effect on the swelling rate of the scaffold (*p* > 0.05). When the content of β-GP was constant, the swelling rate of the scaffold decreased significantly with increasing HA/β-TCP content (*p* < 0.05). **(C)** Degradation performance of the scaffolds. Changes in the β-GP content had no significant effect on the degradation performance of the scaffolds. When the β-GP content was constant, the degradation rate in the 2% HA/β-TCP and 4% HA/β-TCP groups was lower than that in the no-HA/β-TCP group and gradually stabilized after the 14th day.

An ideal scaffold material needs not only high porosity to promote cell adhesion and growth but also sufficient compressive strength to provide mechanical support. However, there is a trade-off between porosity and compressive strength, and the two need to be balanced to achieve the best results. In this study, when HA/*β*-TCP was not added, the pores of the 12% *β*-GP scaffold changed from lamellar to honeycomb with the change in the interaction between the CS molecule and *β*-GP. The honeycomb structure significantly reduced the porosity of the scaffold but significantly increased the compressive strength (*p* < 0.05). When 2% HA/*β*-TCP was added, there was no significant difference in the porosity of the scaffolds among the different *β*-GP content groups (*p* > 0.05). The compressive strength of the scaffolds in the 12% β-GP group was significantly greater than that in the other groups (*p* < 0.05). When 4% HA/β-TCP was added, the compressive strength of the scaffold increased significantly with increasing β-GP content, but the porosity decreased with increasing β-GP content (*p* < 0.05). However, when β-GP was increased to 14%, the porosity increased, and the compressive strength decreased. According to the above results, when the *β*-GP content was 12%, the compressive strength of the scaffold was significantly increased (*p* < 0.05), and satisfactory porosity was maintained (Figure 4A).

The scaffold should first be able to support the adhesion and proliferation of canine PDLSCs and provide external nutrients to the cells through appropriate pores. Therefore, after measuring the relationship between porosity and compressive strength, combined with the pore size distribution, a stable honeycomb pore structure of the scaffold was confirmed when 12% *β*-GP was added. The addition of 2% HA/*β*-TCP increased both the roughness and the compressive strength of the scaffold. At 12% *β*-GP, the porosity of the 4% HA/β-TCP content group was less than 60%, significantly lower than that of the 2% HA/*β*-TCP content group. Therefore, the scaffold prepared with 12% *β*-GP and 2% HA/β-TCP was most in line with practical needs.

### Results of the swelling performance test

Swelling performance reflects the hydrophilicity of the scaffold material and represents the ability of the material to absorb body fluid or blood after implantation and promote wound healing. When the HA/β-TCP content was constant, the β-GP content had no significant effect on the swelling rate of the scaffold (*p* > 0.05). When the content of β-GP was constant, the swelling rate of the scaffold decreased significantly with increasing HA/β-TCP content (*p* < 0.05; Figure 4B). After the 14% *β*-GP-4% HA/β-TCP content group was soaked in liquid water for 24 h, removed, and weighed, it was observed that the support was loose, the structure was damaged, the scaffold could not be gripped. The swelling ratio of this sample was not included in the statistical analysis.

### Degradation performance of the scaffolds

Degradation performance is an important property of materials that affects tissue regeneration at damaged sites and indicates the stability of the scaffold structure at a given time. The test results showed that weight loss occurred in all groups by the 4th day, and the degradation rate of the scaffolds increased gradually with time. Changes in the *β*-GP content had no significant effect on the degradation performance of the scaffolds. When the β-GP content was constant, the degradation rate in the 2% HA/β-TCP and 4% HA/β-TCP groups was lower than that in the no-HA/β-TCP group and gradually stabilized after the 14th day (Figure 4C). During the degradation time, the shape of the scaffolds in the liquid did not change significantly, and the scaffolds could be easily picked up and handled. Only the 14% *β*-GP-4% HA/β-TCP had a loose structure on the 4th day of degradation and was not easy to clamp.

In summary, a gel matrix was formed by the bonding of CS to β-GP, and HA/β-TCP particles were added to the matrix. After freeze-drying, a three-dimensional scaffold with an interconnected pore structure was formed. After comparing the physical properties of the scaffolds with different formulations, it was shown that scaffolds prepared with 2% CS, 12% β-GP and a 2% HA/*β*-TCP ratio had a honeycomb pore structure. Approximately 60% of the pores had diameters between 300 and 600 μm, and the porosity was 64.56% ± 2.48%, which was conducive to cell adhesion and proliferation. Moreover, the compressive strength of the scaffold (0.44 ± 0.09 MPa) was balanced with the porosity, which provided a certain mechanical strength for the scaffold, maintained the scaffold structure, and enabled the scaffold to resist pressure from the surrounding tissues. Moreover, the scaffold had good swelling hydrophilicity, was able to absorb additional nutrients, and gradually degraded over time.

Therefore, considering the scaffold performance, scaffolds were prepared with 2% CS, 12% *β*-GP, and 2% HA/β-TCP for subsequent tests.

### Cell proliferation on the scaffold

**Figure 5:**
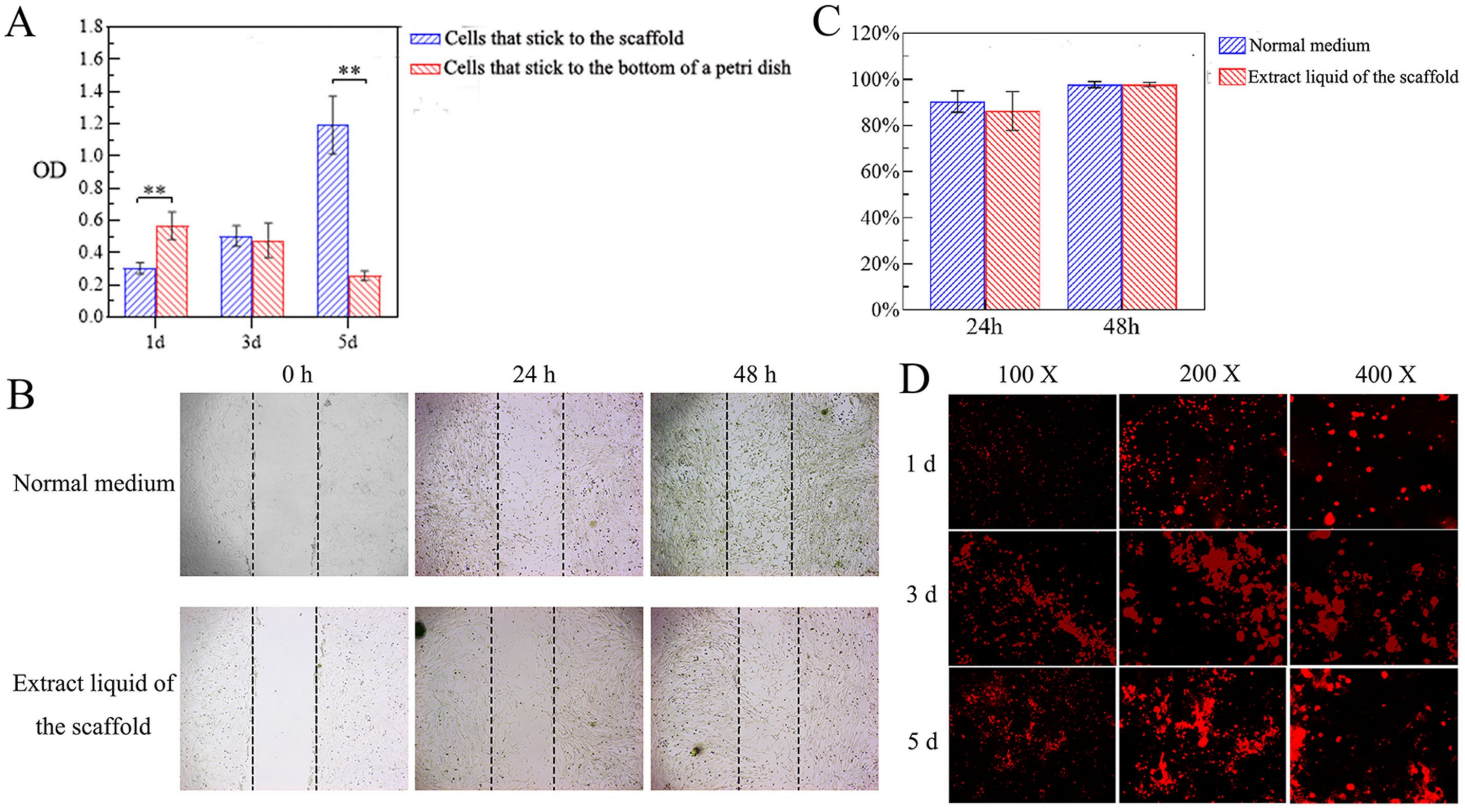
Cytotoxicity of scaffolds. **(A)** Cell proliferation on the scaffold. On the 1st day after the inoculation of canine PDLSCs, the number of cells attached to the bottom of the petri dish was significantly greater than that attached to the scaffold (*p* < 0.05). On Day 3, there were more cells attached to the scaffold than to the bottom of the dish, but the difference was not significant. On the 5th day, the number of cells attached to the scaffold and their proliferation were significantly greater than on the bottom of the Petri dish (*p* < 0.05). **(B,C)** Effects of the scaffold extract liquid on cell migration. At 48 h, the migrating cells were observed to cover almost the entire scratched area. According to the quantitative analysis of the scratch area, there was no significant difference in the cell migration area between the experimental group and the control group at 24 h or 48 h. **(D)** Adhesion and growth of cells on scaffolds. On the 3rd day, the number of cells attached to the scaffold had gradually increased, and the scaffold was moved to a new well to continue to observe the cell growth. On the 5th day, a large number of cells with red fluorescence could be observed, and the red fluorescence was aggregated into clumps.

The proliferative activity of canine PDLSCs on the scaffold was evaluated by CCK-8, and the proliferation of canine PDLSCs on the scaffold increased with time. On the 1st day after the inoculation of canine PDLSCs, the number of cells attached to the bottom of the petri dish was significantly greater than that attached to the scaffold (*p* < 0.05). On Day 3, there were more cells attached to the scaffold than to the bottom of the dish, but the difference was not significant. On the 5th day, the number of cells attached to the scaffold and their proliferation were significantly greater than on the bottom of the Petri dish (*p* < 0.05; Figure 5A). The results showed that the cells could adhere to the scaffold and that the scaffold had no toxic effect on the canine PDLSCs or promoted their proliferation.

### Effects of the scaffold extract liquid on cell migration

The effect of the scaffold extract liquid on the migration ability of canine PDLSCs was investigated via a scratch test. The migration of the cells in the experimental group and the control group to the scratched area were monitored at 24 h and 48 h (Figure 5B). At 48 h, the migrating cells were observed to cover almost the entire scratched area. According to the quantitative analysis of the scratch area, there was no significant difference in the cell migration area between the experimental group and the control group at 24 h or 48 h (Figure 5C). The results showed that the scaffold extract had no toxic effect on canine PDLSCs and did not affect the migration ability of the cells.

### Adhesion and growth of cells on scaffolds

Canine PDLSCs labeled with CM-Dil were cultured together with the scaffolds. Under fluorescence microscopy, cells that had just been inoculated onto the scaffold were round and evenly dispersed across the scaffold. On the 1st day, some cells adhered to the surface of the scaffold and entered the interior of the scaffold. On the 3rd day, the number of cells attached to the scaffold had gradually increased, and the scaffold was moved to a new well to continue to observe the cell growth. On the 5th day, a large number of cells with red fluorescence could be observed, and the red fluorescence was aggregated into clumps (Figure 5D). These findings further indicated that the cells attached to the scaffold and exhibited good cell proliferation.

### *In vivo* biocompatibility test results

The results of basic physiological index monitoring and biochemical examination of the test dogs showed that the test dogs were in good health before and 21 days after the subcutaneous implantation of the tissue engineering scaffolds and scaffolds; additionally, basic indicators such as temperature, respiration, heart rate and pulse were normal, and ALT, AST and CREA were all within the normal reference ranges. There were no complications or adverse events during the healing process. The results showed that the tissue engineering scaffolds and scaffolds exhibited no obvious hepatotoxicity or nephrotoxicity, so follow-up studies could be carried out.

**Figure 6:**
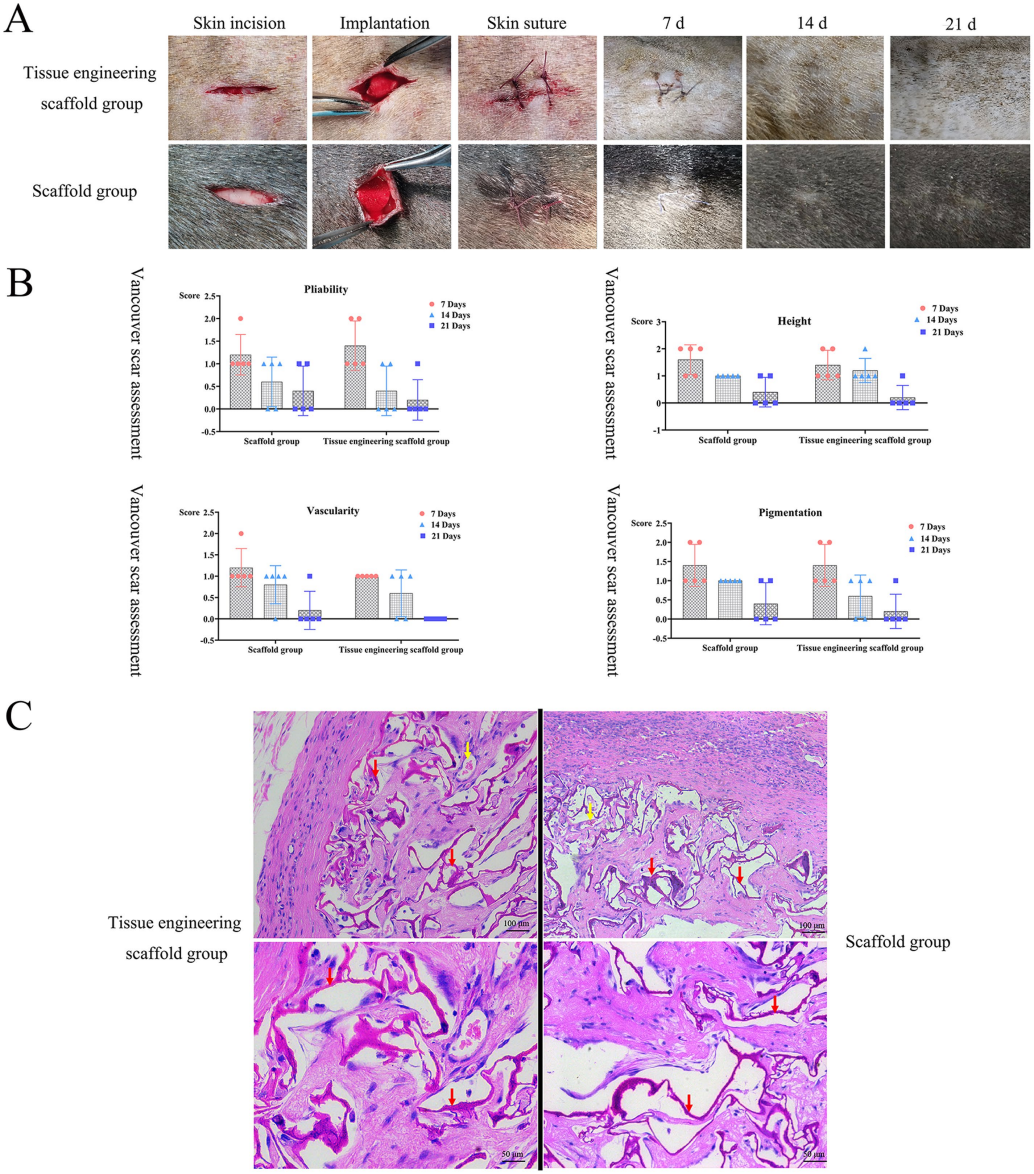
*In vivo* biocompatibility test results. **(A,B)** After the two groups of scaffolds were implanted into the subcutaneous tissue, the healing process and the speed of the wound were not affected, and blood circulation at the implantation site was not affected. **(C)** There was no obvious inflammatory cell infiltration around the scaffolds, new capillaries could be observed, and there was no pathological damage, such as tissue necrosis or hyperplasia, around the scaffolds. Both groups of scaffolds had good histocompatibility *in vivo*. Red arrows indicate remaining scaffolds, yellow arrows indicate blood vessels.

The wound healing process was evaluated on the 7th, 14th and 21st days after surgery. On the 7th day, the surgical wound anastomosis was good, there was no visible tissue gap, linear scars had formed at the wound site, the tissue was grayish brown or reddish-brown in color and deeper than the surrounding skin, and the tissue engineering scaffolds and scaffolds showed no significant effect on the healing of the wound. On the 14th day, the scar tissue had degenerated, the wound was soft, the surrounding color was pink or normal, and the wound color was lighter than or similar to the surrounding skin color. On the 21st day, the skin wounds had healed completely without exudation, redness or obvious inflammatory reactions (Figure 6A). According to assessment by the Vancouver Scar Score, with increasing healing time, the pliability, height, vascularity and pigmentation scores gradually decreased to close to those of normal skin. The scores of the tissue engineering scaffolds were generally lower than those of the scaffolds (Figure 6B). After the two groups of scaffolds were implanted into the subcutaneous tissue, the healing process and the speed of the wound were not affected, and blood circulation at the implantation site was not affected.

When tissue engineering scaffolds are implanted under the skin, there is a dynamic interaction process between the tissue engineering scaffold and the subcutaneous tissue. The tissue engineering scaffolds and other scaffolds both showed good compatibility with subcutaneous tissue. On the 21st day, the scaffolds in both groups had gradually degraded, and the structures were loose. At this time, the surrounding tissues were slowly growing into the scaffolds, but there was still a blank area around the scaffolds. There was no obvious inflammatory cell infiltration around the scaffolds, new capillaries could be observed, and there was no pathological damage, such as tissue necrosis or hyperplasia, around the scaffolds (Figure 6C). Both groups of scaffolds had good histocompatibility *in vivo*.

### Evaluation of the effectiveness of periodontal defect repair and regeneration

In strict accordance with the requirements of single-wall bone defect model construction, a canine first molar bone defect model was generated, and tissue engineering scaffolds (Supplementary material S1A) and scaffolds (Supplementary material S1B) were transplanted into the periodontal defect site, while the control group (Supplementary material S1C) did not undergo any transplantation.

**Figure 7:**
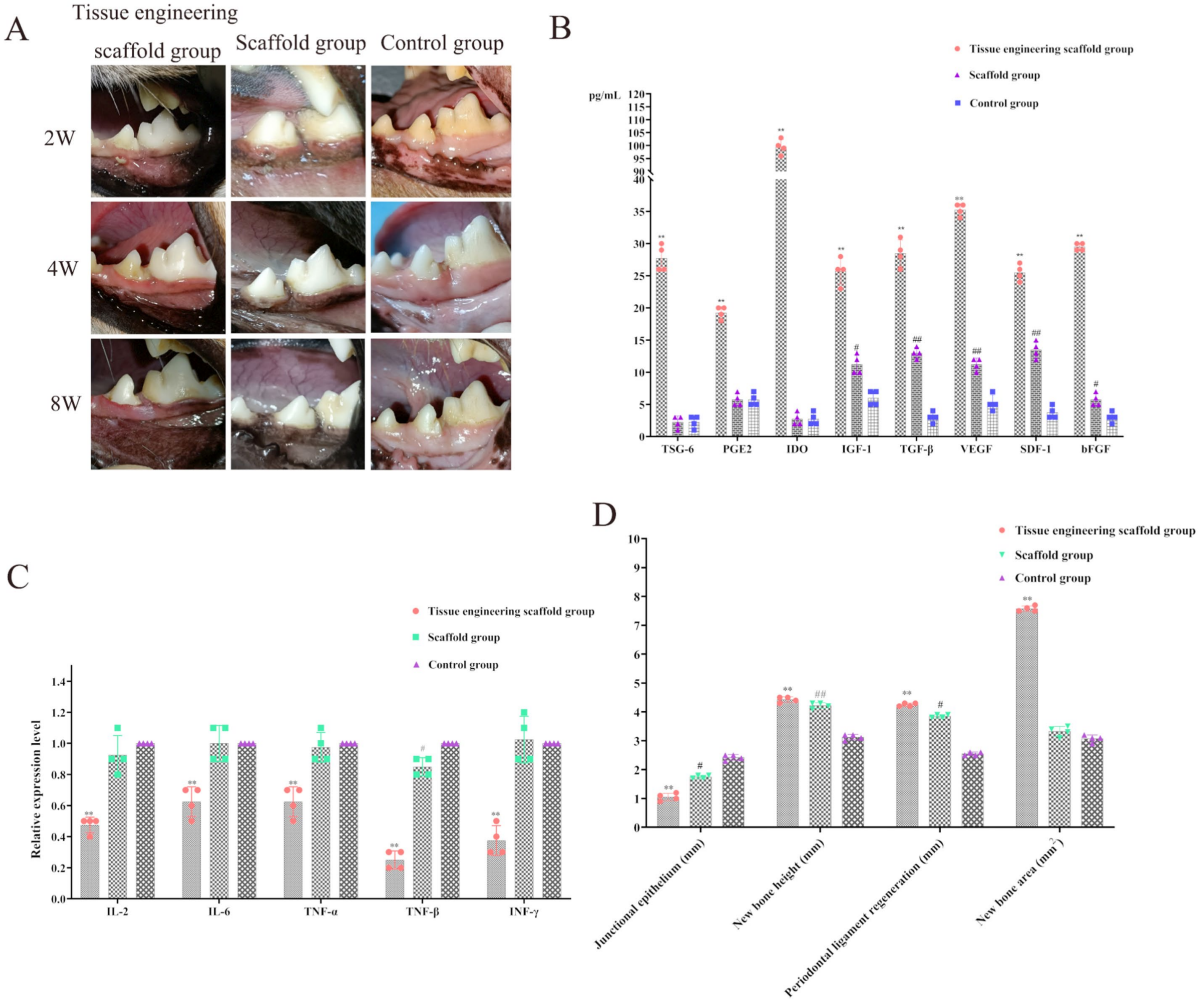
Evaluation of the effectiveness of periodontal defect repair and regeneration. **(A)** The gingival epithelium covered the whole wound in both the tissue engineering scaffold group and the scaffold group, while the periodontal soft tissue in the control group was still concave at the defect site. **(B)** The levels of TSG-6, PGE2, IDO, IGF-1, TGF-β, VEGF, SDF-1 and bFGF in the tissue engineering scaffold group were significantly greater than those in the scaffold group and control group (*p* < 0.01). The levels of IGF-1, TGF-β, VEGF, SDF-1 and bFGF in the scaffold group were significantly greater than those in the control group (*p* < 0.01, *p* < 0.05). **(C)** IL-2, IL-6, TNF-*α*, TNF-β and INF-*γ*, were significantly decreased after implantation of the tissue-engineered scaffold containing PDLSCs (*p* < 0.01). TNF-β expression in the scaffold group was significantly lower than that in the control group (*p* < 0.05). **(D)** The results for the junctional epithelium, new bone height, new bone area and periodontal ligament regeneration.

Model dogs with periodontal defects were treated with GTR. At the second week after surgery, the wounds in all the operation groups were closed, and the scaffolds could stably fill the defect area without protruding from the wound. The sutures in the oral cavity of some dogs were not fully absorbed, and gingival bleeding occurred in all groups during periodontal probing. At the 4th week after surgery, the sutures in the mouth of each group were completely absorbed, and the gums of the tissue engineering scaffold group and the scaffold group surrounded and covered the teeth, while the periodontal soft tissues in the control group had collapsed into the defect area and formed a depression. At the 8th week after surgery, the gingival epithelium covered the whole wound in both the tissue engineering scaffold group and the scaffold group, while the periodontal soft tissue in the control group was still concave at the defect site (Figure 7A). In addition, no significant inflammatory reactions, such as tissue edema, necrosis, or suppuration, occurred in the surgical site or surrounding tissues within 8 weeks after surgery. In summary, the use of scaffolds in GTR can support the defect area, prevent the junctional epithelium above the material from growing into the defect area, create space for the repair and regeneration of the defect area, and stabilize the gingival tissue flap.

Moreover, the levels of TSG-6, PGE2, IDO, IGF-1, TGF-*β*, VEGF, SDF-1 and bFGF in the tissue engineering scaffold group were significantly greater than those in the scaffold group and control group (*p* < 0.01). The levels of IGF-1, TGF-β, VEGF, SDF-1 and bFGF in the scaffold group were significantly greater than those in the control group (*p* < 0.01; *p* < 0.05; Figure 7B). After the implantation of tissue-engineered scaffolds containing PDLSCs, the PDLSCs can secrete factors to promote the repair and growth of bone tissue and other tissues, increase immunosuppression and reduce the inflammatory response. In addition, the scaffolds can stimulate the expression of related factors in surrounding tissues to a certain extent. This process also contributes to periodontal tissue repair and growth (Figure 7B).

Analysis of the three groups of related immune factors revealed that the levels of inflammatory factors, such as IL-2, IL-6, TNF-*α*, TNF-*β* and INF-*γ*, were significantly decreased after implantation of the tissue-engineered scaffold containing PDLSCs (*p* < 0.01), further demonstrating that PDLSCs can secrete immunosuppressive factors. Thus, the tissue engineered scaffold was able to exert immunomodulatory effects, reduce the inflammatory response, reduce transplant rejection, and increase the duration of cell function (Figure 7C). Moreover, TNF-β expression in the scaffold group was significantly lower than that in the control group (*p* < 0.05), indicating that the scaffold in this study inhibited the expression of TNF-β in surrounding tissues, which is highly important for reducing the inflammatory response and accelerating the process of tissue repair (Figure 7C).

The results for the junctional epithelium, new bone height, new bone area and periodontal ligament regeneration were as follows:

Junctional epithelium: The binding epithelial volumes in the scaffold group, scaffold group and control group were 1.05 ± 0.22 mm, 1.75 ± 0.17 mm and 2.42 ± 0.14 mm, respectively. There were significant differences between the tissue engineering scaffold group and the control group (*p* < 0.01) and between the scaffold group and the control group (*p* < 0.05; Figure 7D).

New bone height: The new alveolar bone heights in the tissue engineering scaffold group, scaffold group and control group were 4.43 ± 0.22 mm, 4.22 ± 0.23 mm and 3.13 ± 0.26 mm, respectively. There were significant differences between the tissue engineering scaffold group and the control group (*p* < 0.01) and between the scaffold group and the control group (*p* < 0.01; Figure 7D).

New bone area: The new alveolar bone areas in the tissue engineering scaffold group, scaffold group and control group were 7.76 ± 0.24 mm^2^, 3.33 ± 1.12 mm^2^ and 3.08 ± 0.15 mm^2^, respectively. The alveolar bone surface area in the tissue engineering scaffold group was significantly greater than that in the other groups (*p < 0.01*), and the new bone area in the scaffold group was greater than that in the control group, but the difference was not significant (*p* > 0.05; Figure 7D).

Periodontal ligament regeneration: The periodontal ligament regeneration volumes in the tissue engineering scaffold group, the tissue engineering scaffold group and the control group were 4.25 ± 0.33 mm, 3.85 ± 0.27 mm and 2.55 ± 0.19 mm, respectively. There were significant differences between the tissue engineering scaffold group and the control group (*p < 0.01*) and between the scaffold group and the control group (*p* < 0.05; Figure 7D).

**Figure 8:**
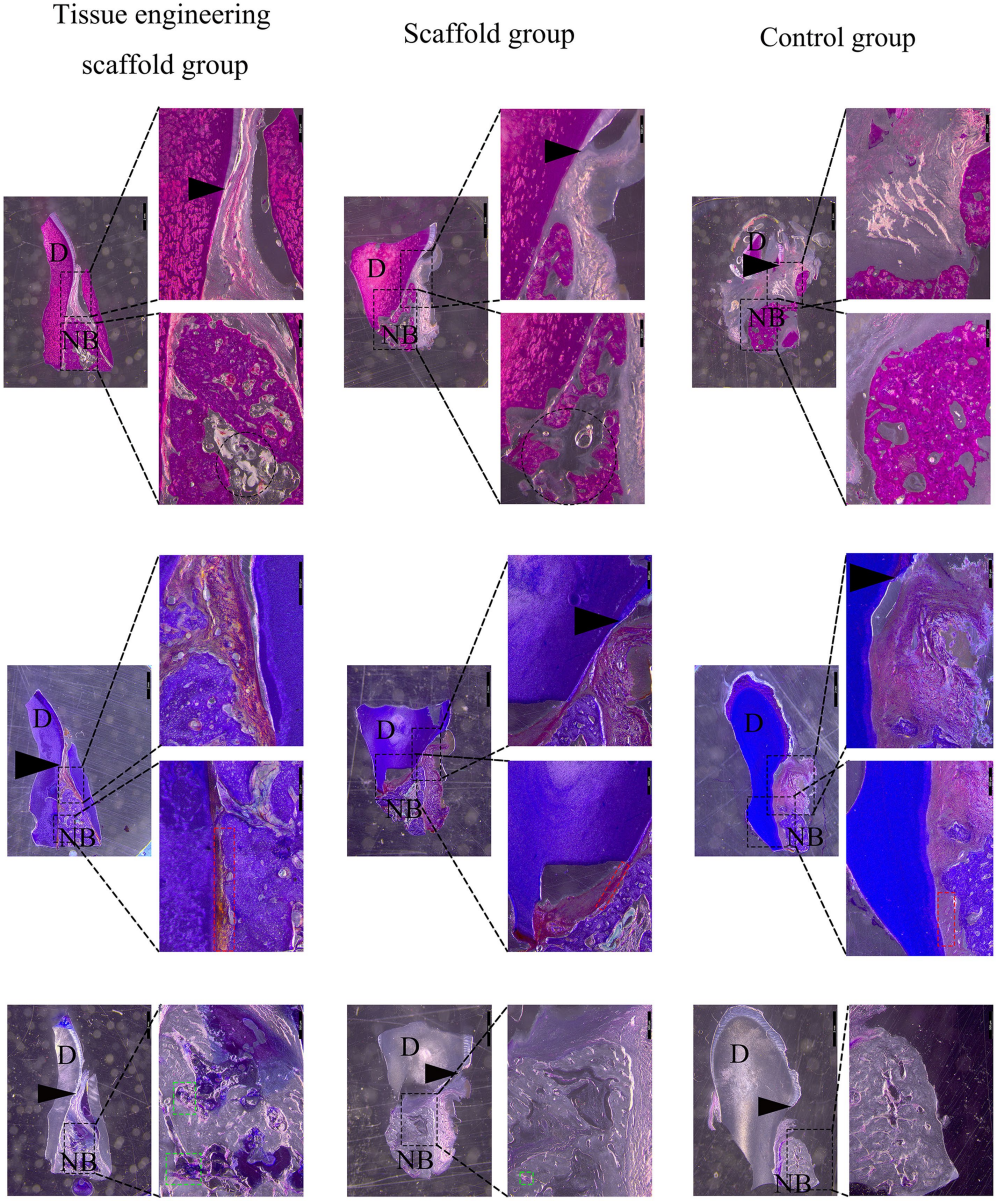
Evaluation of the effectiveness of periodontal defect repair and regeneration. At the 8th week after the operation, the tissue from the dog transplantation site was collected, and hard tissue sections 8 ~ 10 μm in thickness were obtained by grinding the slices. The results from top to bottom were HE staining, Masson staining and toluidine blue staining. D represents teeth, NB represents new alveolar bone, the black arrow points to the enamel bone boundary, the red box represents periodontal tissue, and the green box represents osteoblasts. A black round frame indicates a residual support.

At the 8th week after the operation, the tissue from the dog transplantation site was collected, and hard tissue sections 8 ~ 10 μm in thickness were obtained by grinding the slices. No infiltration of immune cells such as mast cells and macrophages was found in the section. The results were as follows:

Tissue engineering scaffold group: New alveolar bone was found in the defect site within 6 mm of the enamel bone boundary. Osteoblasts were found in the lacunae of the alveolar bone, and scaffold remnants were found in the area empty of new bone. Osteoblasts were observed around the scaffold, and some of the scaffold was replaced by new bone. Neonatal periodontal membrane fibers formed between the alveolar bone and cementum and attached to both sides. A gingival furrow was formed between the gingival junctional epithelium and tooth enamel to maintain a normal level of periodontal attachment (Figure 8).

Scaffold group: New alveolar bone was found in the defect site within 6 mm of the enamel bone boundary. However, there were more blank areas in the new alveolar bone than in the tissue engineering scaffold group, and the bone density was lower. Scaffolds were found in the area empty of new bone, and other scaffolds were replaced by new bone. Moreover, neonatal periodontal membrane fibers formed between the alveolar bone and the cementum, but because the new alveolar bone was incomplete and the edges were uneven, the periodontal membrane fibers were not tightly attached. A gingival furrow was formed between the gingival junctional epithelium and tooth enamel to maintain a normal level of periodontal attachment (Figure 8).

Control group: New alveolar bone was found in the defect site within 6 mm of the enamel bone boundary. However, the coronal height and horizontal direction of the new alveolar bone were less than those of the tissue engineering scaffold group and scaffold group. The soft tissue above the alveolar bone collapsed into the defect area and extended between the alveolar bone and the cementum, occupying the space needed for bone formation and periodontal membrane fiber attachment. Moreover, the adhesion of the gingival junctional epithelium decreased, and the gingival groove deepened (Figure 8).

## Discussion

PDLSCs play important roles in maintaining the nutritional supply of teeth, maintaining the balance of the microenvironment and repairing damaged tissues. It is very important to obtain sufficient seed cells and to maintain the normal biological functions of the cells. Establishing a complete process for isolating and cultivating canine PDLSCs can increase the efficiency of the entire experimental study and lay a foundation for subsequent studies (12). In the present study, canine PDLSCs isolated by enzyme digestion combined with the tissue block method were used as new seed cells for tissue engineering research. The resulting PDLSCs had the biological characteristics of mesenchymal stem cells and were successfully isolated and cultured *in vitro*, and a variety of identification results showed that the PDLSCs exhibited self-division and continuous expansion. Moreover, the PDLSCs exhibited multidirectional differentiation ability under appropriate induction conditions, indicating that PDLSCs could participate in the repair and regeneration of native tissue. Hakki et al. reported that there was no statistically significant difference in the expression of osteogenic genes between PDLSCs and dental pulp stem cells, while PDLSCs showed higher expression of type *Ι* collagen (13). Chen et al. used PDLSCs combined with GTR to treat periodontal defects and reported that the alveolar bone height increased in both the cell group and the control group but that the difference was not significant. However, it was proven that PDLSCs were safe for treating periodontal defects, and the combination of cell therapy with scaffolds was proposed for effective periodontal regeneration (14). Therefore, in the present study, canine PDLSCs were selected as seed cells and combined with scaffolds to create tissue engineering scaffolds for repairing periodontal defects. This study showed that canine PDLSCs can secrete related growth factors and immune modulator factors, which play an important role in tissue repair and immune regulation, thereby reducing inflammatory response, reducing transplantation rejection, increasing the time for cells to play their role, and promoting periodontal tissue repair.

In recent years, combinations of stem cells, biomaterials and biochemical factors have been used to improve the repair and regeneration of periodontal tissue to restore and enhance tissue function, providing new ideas for the treatment of periodontal disease. A cell suspension prepared *in vitro* can be directly injected into the injured site, and the advantages of this method include simplicity and minimal invasiveness. However, after injection, there are problems such as insufficient cell supply, the spread of injected cells to surrounding healthy tissues, and the loss of control of cell fate (15). Tissue engineering scaffolds constructed from stem cells and scaffolds can stabilize the cells. According to the theory of tissue engineering, culturing stem cells with scaffolds is expected to promote greater new bone formation. Canine PDLSCs are the most directly relevant potential seed cells for periodontal tissue regeneration. According to histological studies of human periodontal regeneration, the use of biomaterials in periodontal therapy can promote periodontal regeneration (16). Saito et al. treated patients with periodontal defects larger than 3 mm via guided regeneration combined with rhFGF-2 and DBBM; the clinical attachment level of patients in the experimental group improved, and the bone filling volume in the experimental group was significantly greater than that in the control group (17).

Chitosan (CS) is a biodegradable and biocompatible material that is widely used in research on wound healing because of its good adhesion and antibacterial properties (18). CS is generally insoluble in water and organic solvents, but in 1 M acidic solution, the amine group is dissociated into a cationic state (19). Under these conditions, when *β*-GP is added to the CS solution, the phosphate anion can form an ion complex with the CS amine group through ion coupling (20). After freeze-drying, a porous structure can be formed, and CS scaffolds with interconnected pores can be prepared. The results of this study showed that when the content of *β*-GP was 6% ~ 14%, it could crosslink with CS to form a stable gel matrix. However, the mechanical properties of CS scaffolds are not strong, and their ability to guide osteogenesis is inadequate for periodontal tissue repair. HA/*β*-TCP is a biphasic calcium phosphate bone substitute material composed of HA and β-TCP that can release calcium and phosphorus ions and has good biocompatibility, bone conductivity, and bone induction ability and other advantageous properties (21). Accordingly, when HA/β-TCP particles were dispersed into the gel matrix, the gelation time was decreased, and the gel state remained stable. After the scaffold was freeze-dried in the gel state, the pores of the scaffold were used as the space for nutrient exchange, cell migration and other activities. The results of this study showed that at the same β-GP concentration, the addition of HA/β-TCP enhanced the compressive strength of the scaffold, but the porosity decreased with increasing HA/β-TCP concentration. The porosity of the scaffold in the 4% HA/β-TCP content group was significantly lower than that in the 2% HA/β-TCP content group. In a study on the properties of glass–ceramic scaffolds, a linear negative correlation between the porosity of the scaffolds and the compression modulus was also reported (22). However, the swelling of the 2% HA/β-TCP stent group was greater than that of the 4% HA/β-TCP group, and the addition of 2% HA/β-TCP to the scaffold was more suitable for the actual demands of low-load applications such as alveolar bone repair. Moreover, this study showed that the scaffold stimulated the expression of related factors (including growth factors and immunosuppressive factors) in the surrounding tissues, which was conducive to the repair and growth of periodontal tissues.

The biocompatibility of scaffold materials is manifested mainly in cell adhesion, growth, migration and proliferation on the scaffold. The adaptive response between cells and scaffolds is highly important for subsequent transplantation therapy. *In vitro*, when cells began to exhibit contact inhibition, the scaffold provided additional space for canine PDLSCs to survive, and the proliferation of canine PDLSCs attached to the scaffold became significantly greater. The tissue engineering scaffold extract had no toxic effect on canine PDLSCs and did not affect cell migration. Moreover, cells labeled with red fluorescence adhered to the scaffold and proliferated continuously, indicating that the tissue engineering scaffold constructed in the present study had satisfactory biocompatibility *in vitro* and could be cocultured with cells to form a cell-scaffold complex. Moreover, when the cell-scaffold complex was implanted in the subcutaneous tissue in this experiment, no adverse effects on the liver or kidney function of the dogs were observed within 21 days and the healing of surgical wounds was not affected. Histopathological analysis revealed that after implantation for 21 days, the tissue engineering scaffold became thin and loose in structure, with surrounding tissues growing into the scaffold; moreover, there were small new blood vessels and fibroblast-like cells inside the scaffold and no obvious inflammatory cell infiltration was observed around the scaffold, indicating good biocompatibility *in vivo*.

During transplantation, the tissue engineering scaffold was observed to fit closely to the edge of the periodontal defect and to exhibit good swelling. The surrounding blood penetrated into the scaffold, which enabled wound healing and provided nutrients for cells, and the scaffold gradually degraded over time. The wound healed well and exhibited no tissue edema, necrosis, suppuration or other inflammatory reactions. The histopathological results showed that the size of the new periodontal membrane and the height and area of the new bone were significantly greater when the tissue engineering scaffold was placed at the defect for guided regeneration than in the control defects. New bone formed around the scaffold, indicating that the scaffold maintained space for new bone formation. In contrast, guided regeneration was not performed in the control group. As the bone growth rate was slower than that of epithelial tissue, the gingiva combined with the epithelium and connective tissue extended into the defect area, occupying the position where new bone formation was needed and thus limiting the growth space for new bone. Therefore, guided regeneration by placing tissue engineering scaffolds in the periodontal defect area can effectively block the binding of epithelium outside the periodontal defect area, preserve space for the formation of new bone, stabilize the gingival tissue flap to prevent collapse into the periodontal defect area, and prevent the plaque from forming in the defect.

Masson staining showed that the new periodontal membrane fibers in the tissue engineering scaffold group were attached to the new bone, while the periodontal membrane fibers in the control group were not firmly attached. Toluidine blue staining showed osteoblasts around the new bone in the tissue engineering scaffold group but the number of osteoblasts inside the tissue engineering scaffold was smaller, while no osteoblasts were observed in the middle of the new bone in the control group. Therefore, it is speculated that the implanted canine PDLSCs, on the one hand, can differentiate into osteoblasts and periodontal cells to promote periodontal regeneration under the influence of the periodontal microenvironment; on the other hand, scaffold materials can also induce cell differentiation. In addition, the factors secreted by canine PDLSCs can inhibit transplantation rejection and promote periodontal tissue repair. HA/*β*-TCP is a biphase calcium phosphate, similar to the compounds in natural bone tissue, and studies have confirmed that the addition of HA can not only increase the compression modulus of the scaffold but also provide a larger adhesion surface, thereby increasing cell adhesion, biological activity and proliferation (23, 24). *β*-TCP is also widely used in bone defect repair research and has good biocompatibility and bone-guiding effects (25). Moreover, β-GP is one of the main components of osteogenic induction fluid and can provide the phosphorus ions necessary for bone tissue precipitation, thereby accelerating the calcification of nodules (26). Therefore, the various components of tissue engineering scaffolds may promote the differentiation of canine periodontal stem cells into osteoblasts and periodontal cells and the formation of more new bone and periodontal membranes in the defect area than in the single scaffold group. However, in this study, the new periodontal tissue did not completely fill the periodontal defect area, so it is necessary to continue to improve the support provided by the tissue engineering scaffold and add corresponding cytokines to promote complete periodontal regeneration.

## Conclusion

In the present study, canine PDLSCs isolated by enzyme digestion combined with the tissue block method exhibited fibrous adherent growth and could form clonal colonies with phenotypic characteristics of mesenchymal stem cells, which could differentiate into adipose, cartilage and bone cells. The scaffolds prepared with 2% CS, 12% β-GP and 2% HA/β-TCP had good physical properties and biocompatibility both *in vivo* and *in vitro*. The canine PDLSC tissue engineering scaffold was transplanted into the single wall bone defect of the first mandibular molar tooth of the dog without causing tissue edema, necrosis, suppuration or other inflammatory reactions, and the tissue compatibility was satisfactory. The cell-scaffold complex can increase the content of related growth factors and immunomodulatory factors in tissues, reduce the content of proinflammatory factors, and prevent the growth of binding epithelium into the defect area, thus forming new bone and new periodontal ligaments in the defect area, promoting the repair of periodontal defects, and improving the therapeutic effect of guided regeneration.

## Data Availability

The original contributions presented in the study are included in the article/Supplementary material, further inquiries can be directed to the corresponding author/s.

## References

[1] Marshall MD Wallis CV Milella L Colyer A Tweedie AD Harris S. A longitudinal assessment of periodontal disease in 52 miniature schnauzers. BMC Vet Res. (2014) 10:166. doi: 10.1186/1746-6148-10-166 25179569 PMC4236762

[2] Wallis C Holcombe LJ. A review of the frequency and impact of periodontal disease in dogs. J Small Anim Pract. (2020) 61:529–40. doi: 10.1111/jsap.13218 32955734

[3] Tamura K Tokuzen-Tai M Siddiqui YD Tamura-Naito H Nagahara Y Hatanaka-Takeuchi K. Estimation of periodontal pocket surface area in small to medium dogs: a proof-of-concept study. BMC Vet Res. (2022) 18:13. doi: 10.1186/s12917-021-03116-0, PMID: 34980120 PMC8722143

[4] Chapple IL. Time to take periodontitis seriously. BMJ. (2014) 348:g2645. doi: 10.1136/bmj.g2645 24721751

[5] Vaquette C Pilipchuk SP Bartold PM Hutmacher DW Giannobile WV Ivanovski S. Tissue engineered constructs for periodontal regeneration: current status and future perspectives. Adv Healthc Mater. (2018) 7:e1800457. doi: 10.1002/adhm.201800457 30146758

[6] Kantarci A. Biological basis of periodontal regeneration. Dent Clin N Am. (2022) 66:1–9. doi: 10.1016/j.cden.2021.08.001 34794547

[7] Zhu W Zhang Q Zhang Y Cen L Wang J. PDL regeneration via cell homing in delayed replantation of avulsed teeth. J Transl Med. (2015) 13:357. doi: 10.1186/s12967-015-0719-2, PMID: 26572489 PMC4647325

[8] Soldatos NK Stylianou P Koidou VP Angelov N Yukna R Romanos GE. Limitations and options using resorbable versus nonresorbable membranes for successful guided bone regeneration. Quintessence Int. (2017) 48:131–47. doi: 10.3290/j.qi.a37133 27834419

[9] Ouchi T Nakagawa T. Mesenchymal stem cell-based tissue regeneration therapies for periodontitis. Regen Ther. (2020) 14:72–8. doi: 10.1016/j.reth.2019.12.011, PMID: 31970269 PMC6962327

[10] Tomokiyo A Wada N Maeda H. Periodontal ligament stem cells: regenerative potency in periodontium. Stem Cells Dev. (2019) 28:974–85. doi: 10.1089/scd.2019.0031 31215350

[11] Ueberschaer M Endres M Wachtel N Oehlschlägel F Thorsteinsdottir J Schichor C. A prospective randomized comparison of functional and cosmetic outcomes of a coronal zigzag incision versus a conventional straight incision pattern for craniotomy. J Neurosurg. (2023) 140:1–8. doi: 10.3171/2023.10.JNS231813, PMID: 38157520

[12] Amato M Santonocito S Viglianisi G Tatullo M Isola G. Impact of Oral mesenchymal stem cells applications as a promising therapeutic target in the therapy of periodontal disease. Int J Mol Sci. (2022) 23. doi: 10.3390/ijms232113419, PMID: 36362206 PMC9658889

[13] Hakki SS Kayis SA Hakki EE Bozkurt SB Duruksu G Unal ZS. Comparison of mesenchymal stem cells isolated from pulp and periodontal ligament. J Periodontol. (2015) 86:283–91. doi: 10.1902/jop.2014.140257, PMID: 25325708

[14] Chen X Wu G Feng Z Dong Y Zhou W Li B. Advanced biomaterials and their potential applications in the treatment of periodontal disease. Crit Rev Biotechnol. (2016) 36:760–75. doi: 10.3109/07388551.2015.1035693 26004052

[15] Mooney DJ Vandenburgh H. Cell delivery mechanisms for tissue repair. Cell Stem Cell. (2008) 2:205–13. doi: 10.1016/j.stem.2008.02.005 18371446

[16] Sculean A Nikolidakis D Nikou G Ivanovic A Chapple IL Stavropoulos A. Biomaterials for promoting periodontal regeneration in human intrabony defects: a systematic review. Periodontol. (2015) 68:182–216. doi: 10.1111/prd.12086 25867987

[17] Saito A Bizenjima T Takeuchi T Suzuki E Sato M Yoshikawa K. Treatment of intrabony periodontal defects using rhFGF-2 in combination with deproteinized bovine bone mineral or rhFGF-2 alone: a 6-month randomized controlled trial. J Clin Periodontol. (2019) 46:332–41. doi: 10.1111/jcpe.13086, PMID: 30758076 PMC6899590

[18] Lauritano D Limongelli L Moreo G Favia G Carinci F. Nanomaterials for periodontal tissue engineering: Chitosan-based scaffolds. A systematic review. Nanomaterials (Basel). (2020) 10:605. doi: 10.3390/nano10040605 32218206 PMC7221778

[19] Ou Y Tian M. Advances in multifunctional chitosan-based self-healing hydrogels for biomedical applications. J Mater Chem B. (2021) 9:7955–71. doi: 10.1039/D1TB01363G 34611684

[20] Zang S Dong G Peng B Xu J Ma Z Wang X. A comparison of physicochemical properties of sterilized chitosan hydrogel and its applicability in a canine model of periodontal regeneration. Carbohydr Polym. (2014) 113:240–8. doi: 10.1016/j.carbpol.2014.07.018 25256481

[21] Mishchenko O Yanovska A Sulaieva O Moskalenko R Pernakov M Husak Y. From synthesis to clinical trial: novel bioinductive calcium deficient HA/β-TCP bone grafting nanomaterial. Nanomaterials (Basel). (2023) 13:876. doi: 10.3390/nano13121876, PMID: 37368306 PMC10326822

[22] Baino F Ferraris M Bretcanu O Verné E Vitale-Brovarone C. Optimization of composition, structure and mechanical strength of bioactive 3-D glass-ceramic scaffolds for bone substitution. J Biomater Appl. (2013) 27:872–90. doi: 10.1177/0885328211429193, PMID: 22207602

[23] Barbosa RM Da Rocha DN Bombaldi De Souza RF Santos JL Ferreira JRM Moraes Â. Cell-friendly chitosan-xanthan gum membranes incorporating hydroxyapatite designed for periodontal tissue regeneration. Pharmaceutics. (2023) 15:705. doi: 10.3390/pharmaceutics15020705 36840027 PMC9962096

[24] Filippi M Born G Chaaban M Scherberich A. Natural polymeric scaffolds in bone regeneration. Front Bioeng Biotechnol. (2020) 8:474. doi: 10.3389/fbioe.2020.00474, PMID: 32509754 PMC7253672

[25] Lindner M Bergmann C Telle R Fischer H. Calcium phosphate scaffolds mimicking the gradient architecture of native long bones. J Biomed Mater Res A. (2014) 102:3677–84. doi: 10.1002/jbm.a.35038 24307071

[26] Miranda SC Silva GA Hell RC Martins MD Alves JB Goes AM. Three-dimensional culture of rat BMMSCs in a porous chitosan-gelatin scaffold: a promising association for bone tissue engineering in oral reconstruction. Arch Oral Biol. (2011) 56:1–15. doi: 10.1016/j.archoralbio.2010.08.018 20887975

