# Erratum: Periodontal ligament stem cell tissue engineering scaffolds can guide and promote canine periodontal tissue regeneration

**DOI:** 10.3389/fvets.2025.1590975

**Published:** 2025-03-26

**Authors:** Frontiers Production Office

**Affiliations:** Frontiers Media SA, Lausanne, Switzerland

**Keywords:** periodontal disease, periodontal ligament stem cells, canine PDLSC tissue engineering scaffold, guide periodontal regeneration, periodontal repair

Due to a production error, the incorrect abstract was included in the article.

The correct abstract reads as follows:

**Introduction:** Periodontal disease, including gingivitis and periodontitis, is caused by dental plaque invading the periodontal tissues and is the most common oral disease. The basic treatment methods applied in the clinic can destroy dental plaque, smooth the root surface, and reduce local inflammation, but it is difficult to completely regenerate and rebuild the complex three-dimensional periodontal tissues. The rapid development of periodontal tissue engineering has led to the development of new methods for the treatment of periodontal disease. Periodontal ligament stem cells (PDLSCs) are key seed cells in periodontal tissue engineering, which can provide strong support for tissue regeneration. Meanwhile, an engineering scaffold constructed from biomaterials provides a three-dimensional space for the growth and function of seed cells and can form a tissue engineering complex with the seed cells to repair periodontal tissue, which can guide consequently enable true three-dimensional periodontal structure regeneration and functional restoration.

**Methods:** This study established an effective way to isolate, culture, and identify canine PDLSCs. Using chitosan, β-glycerol phosphate, and biphasic calcium phosphate bone substitute as raw materials, a tissue engineering scaffold with good physical properties was prepared by freeze-drying method. Canine PDLSCs were co-cultured with the scaffolds to prepare canine PDLSC tissue engineering scaffolds with good biocompatibility *in vivo* and *in vitro*.

**Results and discussion:** The canine PDLSC tissue engineering scaffold was transplanted into the single wall bone defect of the first mandibular molar tooth of the dog without causing inflammatory reactions, and the tissue compatibility was satisfactory. The cell-scaffold complex can increase the content of related growth factors and immunomodulatory factors in the tissues, reduce the content of proinflammatory factors, and prevent the growth of binding epithelium in the defect area, thus forming new bone and new periodontal ligaments in the defect area, promoting the repair of periodontal defects, and improving the therapeutic effect of guided regeneration.

The publisher apologizes for this mistake. The original version of this article has been updated.

