# Supplementary figures and images for: Periodontal ligament stem cell tissue engineering scaffolds can guide and promote canine periodontal tissue regeneration
Source: Front Vet Sci. 2024 Oct 9;11:1465879. doi: 10.3389/fvets.2024.1465879 (PMC11496256; doi:10.3389/fvets.2024.1465879)

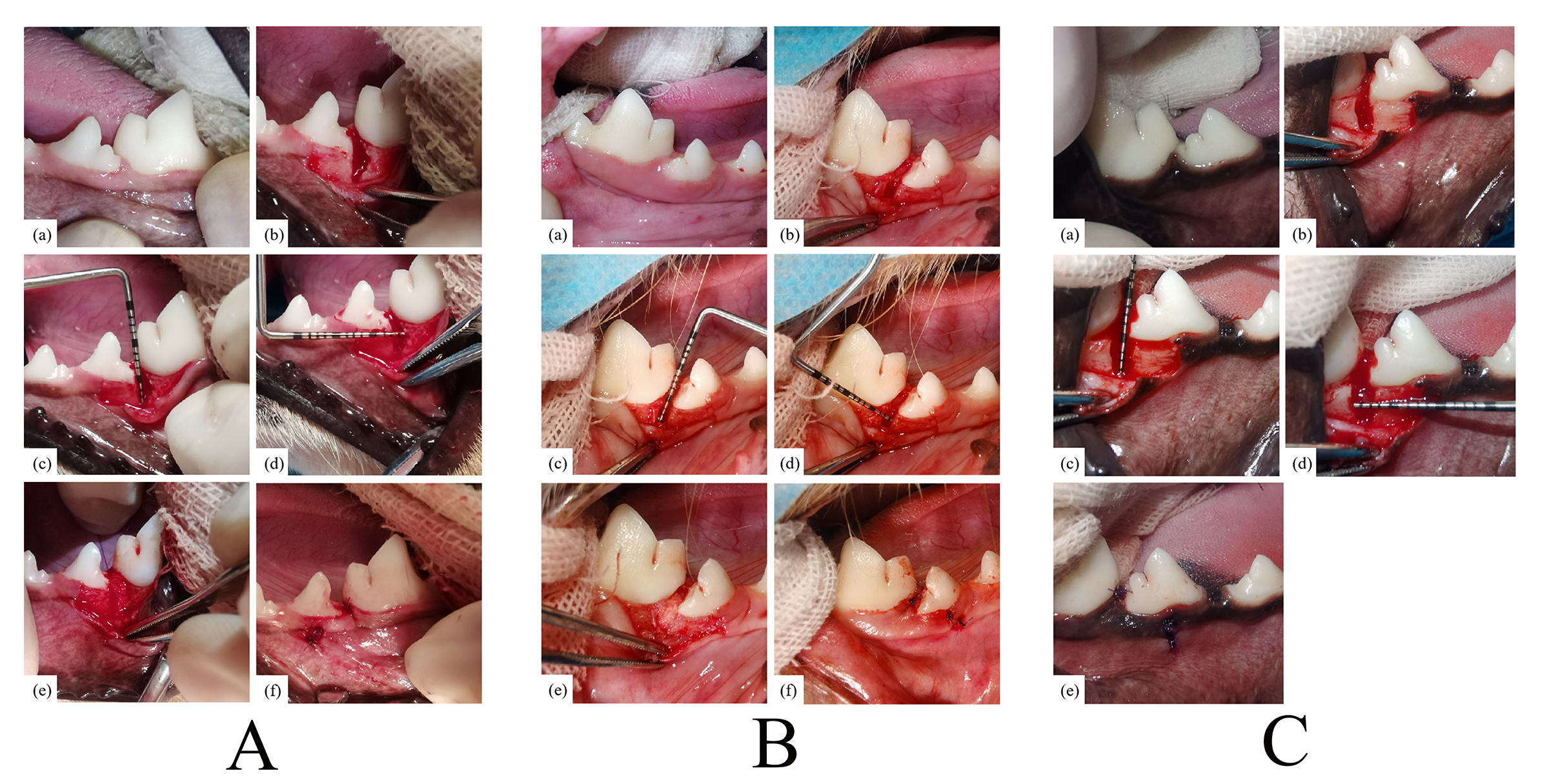

Supplement: Supplementary file 1 [file Image_1.TIF]
